# N1 Repetition-Attenuation for Acoustically Variable Speech and Spectrally Rotated Speech

**DOI:** 10.3389/fnhum.2020.534804

**Published:** 2020-10-29

**Authors:** Ellen Marklund, Lisa Gustavsson, Petter Kallioinen, Iris-Corinna Schwarz

**Affiliations:** Phonetics Laboratory, Department of Linguistics, Stockholm University, Stockholm, Sweden

**Keywords:** N1, repetition-attenuation, neural refractoriness, acoustic variability, spectrally rotated speech, speech processing, repetition-suppression, habituation

## Abstract

The amplitude of the event-related N1 wave decreases with repeated stimulation. This repetition-attenuation has not previously been investigated in response to variable auditory stimuli, nor has the relative impact of acoustic vs. perceptual category repetition been studied. In the present study, N1 repetition-attenuation was investigated for speech and spectrally rotated speech with varying degrees of acoustic and perceptual category variation. In the speech condition, participants (*n* = 19) listened to stimulus trains consisting of either the same vowel exemplar (no variability condition), different exemplars of the same vowel (low variability condition), or different exemplars of two different vowels (high variability condition). In the rotated speech condition, the spectrally rotated counterparts of the vowels were presented. Findings show N1 repetition-attenuation in the face of acoustic and perceptual category variability, but no impact of the degree of variability on the degree of N1 attenuation. Speech stimuli resulted in less attenuation than the acoustically matched non-speech stimuli, which is in line with previous findings. It remains unclear if the attenuation of the N1 wave is reduced as a result of stimuli being perceived as belonging to perceptual categories or as a result of some other characteristic of speech.

## Introduction

The amplitude of the N1 wave^1^ of the event-related potential (ERP) decreases with repeated stimulation (Näätänen and Picton, 1987). In the present study, we test the neural refractoriness hypothesis of N1 repetition-attenuation by examining the impact of acoustic and perceptual category variation on N1 attenuation. If N1 attenuation is a result of neural refractoriness, attenuation should be demonstrated even in the face of acoustic variation, provided stimuli are similar enough that they activate overlapping groups of neurons. We further investigate whether N1 attenuation occurs for both acoustic and perceptual category overlap. Varying the degree of acoustic and perceptual category overlap should in that case lead to varying degrees of N1 repetition-attenuation.

In a typical N1 repetition-attenuation paradigm, a single stimulus is repeated between four and six times with relatively short inter-stimulus intervals (ISIs; 200–1,800 ms), followed by a longer stimulation-free period, typically between 2 and 10 s (Woods and Elmasian, 1986; Rosburg et al., 2010). The N1 amplitude is highest for the first stimulus in such a “train” of repeated stimuli, then gradually or abruptly decreases over the following repetitions to around 30–60% of the response to the first stimulus in the train (Woods and Elmasian, 1986; Yue et al., 2017). The N1 amplitude typically reaches floor levels at the second to third stimulus presentation within a train (Woods and Elmasian, 1986; Rosburg et al., 2010), and then recovers to high levels after the silent inter-train interval.

This amplitude attenuation in N1 has been described using a multitude of terms, for example (short-term) habituation (Woods and Elmasian, 1986; Yue et al., 2017), (repetition-)suppression (Hsu et al., 2016), decrement (Budd et al., 1998; Sörös et al., 2009) and (neuronal) refractoriness (Rosburg et al., 2010). It has been attributed to different perceptual and neuronal phenomena, to some extent evident in the terminology used. Referring to the repetition-attenuation as N1 refractoriness implies that the underlying cause is assumed to be neural refractoriness or adaptation (Budd et al., 1998; Rosburg et al., 2010). This refers to neurons’ inability to respond if a particular stimulus is repeated within a brief-time window, typically attributed to a depletion of releasable neurotransmitters (Sara et al., 2002). The auditory cortex is feature-topically organized, meaning that different sounds give rise to different patterns of activation depending on their spectrotemporal characteristics. The time between repetitions as well as the degree to which these patterns overlap determines the magnitude of N1 attenuation as a result of neural adaptation (May and Tiitinen, 2010). Repetition-attenuation of N1 in a predictive coding framework is instead taken to reflect the stimulus being expected. The discrepancy between the internal model and the physical reality becomes smaller with repeated stimulus presentation, resulting in a decreasing prediction error (Hsu et al., 2016).

N1 repetition-attenuation has been demonstrated both for non-speech stimuli (Rosburg et al., 2010; Leung et al., 2013; Hsu et al., 2016) and for speech stimuli (Yue et al., 2017). The crucial difference between speech and non-speech stimuli is the different types of neural processing they are subjected to; speech stimuli give rise to both, acoustic and linguistic processing while non-speech stimuli—not being perceived as having linguistic meaning—elicit acoustic, but no linguistic processing (e.g., Cheour et al., 2001). Non-speech sounds can of course be associated with words or other linguistic units, just as they can be associated with for example objects or sensations. Importantly, however, the non-speech signal itself is not processed as if it were a speech signal conveying linguistic information. Since non-speech thus comprises more or less all sounds that are not interpreted as carrying linguistic meaning, they can vary greatly in terms of acoustic complexity. This is important to consider when contrasting the processing of speech vs. non-speech.

Directly contrasting N1 attenuation to speech and non-speech stimuli, minor differences have been found. Teismann et al. (2004) found less attenuation of the magnetic counterpart of N1 to a naturally produced vowel in the left hemisphere than in the right hemisphere, but no difference between hemispheres when the stimulus was a sine tone with frequency matching the fundamental frequency of the vowel. This can be attributed to differences in stimulus complexity between conditions, or be related to processing specific to language (Teismann et al., 2004). In contrast, measuring N1 amplitude attenuation at Cz, Woods and Elmasian (1986) report greater attenuation for speech stimuli (vowels and CVC words) than for non-speech stimuli (sine tones and complex tones matching the first three formants of the vowel midpoints), but only at short ISIs (200 ms). For the longer ISIs (700 ms), no difference between conditions was reported (Woods and Elmasian, 1986). As above, possibly the difference in acoustic complexity between conditions impacted the results, but this does not explain the different directions between the two studies. At comparable ISIs, one study shows more N1 attenuation for speech stimuli (200 ms; Woods and Elmasian, 1986), and the other shows less attenuation (190 ms; Teismann et al., 2004).

The present study aims to clarify this discrepancy by comparing the N1 repetition-attenuation to speech and non-speech with comparable acoustic complexity. The non-speech stimuli consist of spectrally rotated speech (Blesser, 1972). A rotated speech signal matches the original speech signal in terms of general acoustic structure and prosodic information but differs in terms of spectral tilt and spectral envelope. Importantly in terms of neural refractoriness, the acoustic difference between two rotated speech tokens can be considered a reasonable approximation of the acoustic difference between the original unrotated versions of the speech tokens. Rotated speech has previously been used as a non-speech control stimulus when attempting to separate processing specific to language from acoustic processing (Scott et al., 2000; Narain et al., 2003; Christmann et al., 2014; Marklund et al., 2018, 2019).

If N1 repetition-attenuation is at least partly caused by neural refractoriness, more attenuation in response to speech stimuli than in response to non-speech stimuli could be an indication of neuronal populations tuned to specific speech sound categories (or characteristics thereof), which with repeated stimulation are depleted of neurotransmitters in a similar way as those sensitive to specific acoustic features. Indicating that this may be the case, mismatch negativity (MMN) has been reported for speech sound category deviations despite the enormous acoustical variation, constituted by 450 vowel tokens uttered by different speakers and no exemplar repetition (Shestakova et al., 2002). Although repetition-attenuation was not explicitly studied or reported in that study, the MMN paradigm is built upon the assumption that repeated stimulation flattens the response to standard stimuli in comparison to the deviant stimuli after only a few presentations, so it seems reasonable to assume some sort of short-term attenuation on speech sound category level. Using magnetic resonance imaging, suppression of activation has been demonstrated in response to repeatedly presented speech stimuli in the superior temporal sulcus (Vaden et al., 2010), an area tied to phonological processing (Hickok and Poeppel, 2016) and processing of other complex non-speech perceptual categories (Leech et al., 2009). This suggests that repetition-attenuation occurs not only on an acoustic level but also on the level of perceptual categories, for example, speech sound categories.

In previous studies on N1 repetition-attenuation of speech and non-speech (Woods and Elmasian, 1986; Teismann et al., 2004), it is not possible to separate the potential impact of category repetition from the impact of acoustic repetition, since stimulus trains have consisted of repeated presentations of a single stimulus. In the present study, N1 repetition-attenuation is investigated for speech and rotated speech with different degrees of acoustic variability across tokens within stimulus trains.

The rationale for using acoustic variability to investigate the neural refractoriness hypothesis of N1 repetition-attenuation rests upon what is known about the organization of the auditory cortex. The auditory cortex is tonotopically (Howard et al., 1996), amplitopically (Pantev et al., 1989), and tempotopically (Herdener et al., 2013) organized, as well as populated both by narrowly tuned and broadly tuned groups of neurons (Kato et al., 2017). This means that even acoustically non-identical sounds are likely to activate overlapping populations of neurons to some extent, provided they are not extremely distinct acoustically. If N1 repetition-attenuation is regarded at least partially as a result of neural refractoriness, it is, therefore, reasonable to posit that it will occur in response to acoustically variable stimuli, albeit to a lesser degree than in response to repeated presentation of a single identical sound. To date, N1 attenuation to acoustically variable stimuli has not been studied extensively, but in one study, attenuation of the N1-P2 complex in response to a 250 Hz tone was demonstrated from the repeated presentation of 8,000 Hz tones (Butler, 1972), supporting this suggestion. Additionally, Hsu et al. (2016) found a successive rebound in N1 attenuation when presenting a series of rising tones. Although the purpose of that study was not to study N1 attenuation to acoustically variable stimuli, the N1 amplitude rebound corresponded to increasing acoustic distance from the first tone in a stimulus train.

In the present study, the acoustic variation in the speech and rotated speech conditions is comparable since the size of the acoustic difference between pairs of rotated speech stimuli approximates that of their non-rotated counterparts. The crucial difference between conditions is that adults do not have perceptual categories in place for spectrally rotated speech, as they typically have never been exposed to it. Therefore, no repetition-attenuation is expected for rotated speech on a category level. Contrasting speech and rotated speech thus makes it possible to isolate acoustic repetition-attenuation from perceptual category repetition-attenuation.

To summarize, we work under the assumption that overlap between stimuli at least partially contributes to N1 repetition-attenuation due to neural refractoriness. This refractoriness has been demonstrated on the level of acoustic processing (Budd et al., 1998; Rosburg et al., 2010) and the level of perceptual category processing (Vaden et al., 2010). We study N1 attenuation to speech and non-speech of comparable acoustic complexity (spectrally rotated speech), varying the acoustic and category overlap within stimulus trains. We label our conditions No Variability (NoVar), Low Variability (LoVar), and High Variability (HiVar), as this reflects the relative variability within this study, although it is worth noting that relative to variability found in natural conversations, the acoustic variability in this study is uniformly very low.

We predict that the degree of N1 repetition-attenuation decreases with increased variability between tokens, regardless of speech type condition (Table 1, hypothesis 1). We hypothesize that the N1 amplitude attenuation will reflect both acoustic and linguistic (speech sound category) overlap, and thus assume an additive effect of acoustic overlap and category overlap. For the two speech type conditions, speech and rotated speech, the predictions therefore depend on the variability condition. In the NoVar condition, a single exemplar is repeated, that is, there is a complete overlap between in both speech type conditions. However, for speech, this constitutes a combined acoustic and category overlap whereas for rotated speech this means acoustic overlap only. Consequently, we predict more repetition-attenuation to be present for speech than for rotated speech in the NoVar condition (Table 1, hypothesis 2a). In the LoVar condition, tokens in a train are different exemplars of the same vowel (in the speech condition) or their rotated counterparts (in the rotated speech condition). In both speech type conditions, there is partial acoustic overlap—roughly equal between speech type conditions—but for speech, there is an additional complete category overlap. Therefore, we predict more repetition-attenuation for speech than for rotated speech also in the LoVar condition (Table 1, hypothesis 2b). In the HiVar condition, tokens within a train are exemplars from two vowel categories (in the speech condition) or their rotated counterparts (in the rotated speech condition). This means less acoustic overlap than in the LoVar condition (in both speech type conditions), as well as less perceptual category overlap in the speech condition. Due to this low degree of overlap in both speech and in rotated speech, we predict little N1 repetition-attenuation in the HiVar condition regardless of speech type condition (Table 1, hypothesis 2c).

**Table 1:** Summary of predictions about the degree of attenuation.

#	Prediction	Data
1	NoVar > LoVar > HiVar	Speech and rotated speech pooled
2a	Speech > Rotated speech	NoVar
2b	Speech > Rotated speech	LoVar
2c	Speech = Rotated speech (no N1 suppression)	HiVar

## Material and Method

### Participants

Participants were 19 native speakers of Swedish (10 females) between 24 and 60 years (mean = 39.0, SD = 10.3). An additional three participants took part in the study but were excluded due to technical failure (*n* = 1), left-handedness (*n* = 1), or hearing impairment (*n* = 1). All included participants were right-handed with no reported hearing impairments. Four participants had besides Swedish an additional first language (Spanish, Persian, Serbian, or Swedish Sign Language). All included participants had completed high school and most of them had a university education (*n* = 18). Written informed consent was given by all participants before the experiment and participants were given movie vouchers as compensation for their participation. The study has been approved by the National Swedish Ethics Board (2019-00685).

### Design and Stimuli

The experiment consisted of 20 blocks across two conditions; 10 speech and 10 rotated speech blocks, alternating in order. The starting block with either a speech or a rotated speech block was counterbalanced across participants. Each block consisted of 24 trains of four stimuli each. Each train was separated by a 4.5 s pause. Half of the trains contained variations of the vowel /e/ and half the vowel /i/. The stimulus trains had three types of variation between stimuli; no, low and high variation (NoVar, LoVar, and HiVar, respectively). A NoVar train repeats the same stimulus exemplar four times. A HiVar train contains four exemplars from a continuum of eight exemplars with an equal acoustic distance between the prototypical vowels /e/ and /i/, two from each side of the vowel continuum. A LoVar train contains four exemplars from only one side of this vowel continuum, either exemplars of /e/ or exemplars of /i/. There were 80 trains per variation condition both in the speech and in the rotated speech condition, resulting in 480 trains in total. The stimulus onset asynchrony (SOA) between train stimuli was 500 ms, with an ISI of 160 ms. At the end of each block, there was a designated pause of 15 s to give the participant the possibility to move without causing artifacts.

The stimuli were first created for use in previous studies (Marklund et al., 2017, 2019). Twelve exemplars of each vowel were recorded in /VC/ contexts by a female native speaker of Swedish in an anechoic chamber. Their consonant context was removed and one exemplar of /e/ and one /i/ were chosen as prototypical vowel stimuli based on auditory and acoustic similarity (fundamental frequency, overall intensity, and duration). These vowels were then used as end-points for acoustic interpolation in six equal steps, resulting in a continuum of eight vowels ranging from /i/ to /e/. The acoustic interpolation was done in Praat 6.0.21 (Boersma, 2001) using a script for creating formant continuums (Winn, 2014).

The stimuli for the rotated speech condition were created by spectrally rotating the vowels in the /i/ to /e/ continuum around a center frequency of 2,301 Hz resulting in eight linguistically non-intelligible sounds but with the same acoustic structure as the original vowels in terms of the fundamental frequency, intensity, and duration. The spectral rotation was accomplished by transforming high-frequency components to low frequencies and vice versa in Mathematica (Wolfram Research Inc., Champaign, IL, USA) using the procedure described in previous studies (Marklund et al., 2017, 2019).

The duration of vowel and rotated vowel tokens was 340 ms. Detailed acoustic descriptions of the speech and rotated speech material can be found in the “additional information and data”^2^.

### Apparatus

The EEG data were collected at a sampling rate of 2,048 Hz with the BioSemi ActiveTwo system (BioSemi, Amsterdam, The Netherlands), using the ActiView 7.06 acquisition software. ActiveTwo amplifiers use a CMS/DRL reference and a digital low pass filter with −3 dB at one-fifth of the sampling rate during recording. We measured ERPs from 32 electrodes plus six external electrodes: two to record at the mastoids and four to control for eye movement. The experiment was run in E-Prime 2.0, presenting the audio stimuli *via* two loudspeakers. The participant was seated at a distance of approximately 90 cm from the loudspeakers which presented the stimuli at about 75 dB SPL measured at the participant’s head (using the smartphone application Decibels). During the experiment, the participant watched a silent animated movie on a separate laptop, displaying the movie at a reduced screen size to minimize eye movement during the recording. On a side table next to the participant, the E-Prime computer was situated, signaling breaks and when the next block was about to begin. The offline data analysis was carried out in MATLAB R2019a (MathWorks Inc., Natick, MA, USA), using the EEGLAB 2019.0 toolbox (Delorme and Makeig, 2004).

### Procedure

The experiment was performed in a quiet test room with good acoustics and sound-shielding double doors. After having received instructions and information about the study, the participant provided their written consent. They were seated in a comfortable chair with armrests, facing the laptop to play the silent movie and the two loudspeakers to present the stimuli. The E-Prime computer on the side indicated when there was a 15 s pause between blocks, interrupting stimulus presentation briefly for the participant to adjust their sitting position and move their body. A numeral countdown warned that breaks were ending to help the participant to resume motionless sitting in time. The experiment excluding setup time took about 56 min, 5 min of which was pause time.

### EEG Preprocessing and Analysis

The continuous EEG raw data from 32 plus six channels was resampled to 500 Hz, band-pass filtered at 1–30 Hz, and re-referenced to the average of the two mastoids. In line with recent studies on N1 repetition-attenuation (Hsu et al., 2016; Yue et al., 2017), 100 ms of the ISI before stimulus onset were used as baseline (stimulus onset was delayed 70 ms relative to file onset to reduce loading-related sound artifacts). The recording was segmented into one epoch per vowel, setting an analysis window of 600 ms (including 100 ms baseline).

Independent component analysis (ICA) was performed on the epoched EEG and evaluated independently by two experimenters who identified the most prominent blink and eye movement components for each participant. The identification of eye artifact components had an interanalyzer agreement of 96%. Artifactual components were removed before continuing with the pre-processing. In addition to the ICA-based artifact removal, any trials with amplitude excursions exceeding ±50 μV were rejected.

The electrode Cz was selected *a priori* for the analysis based on previous similar studies (Woods and Elmasian, 1986; Yue et al., 2017). To test for laterality effects as previously reported (Teismann et al., 2004), C3 and C4 were also selected for analysis. The N1 amplitude was quantified as the average of samples within the time window of 70–150 ms after stimulus onset. This time window was selected based on visual inspection of the grand average waveforms pooled across all conditions.

## Results

**Figure 1:**
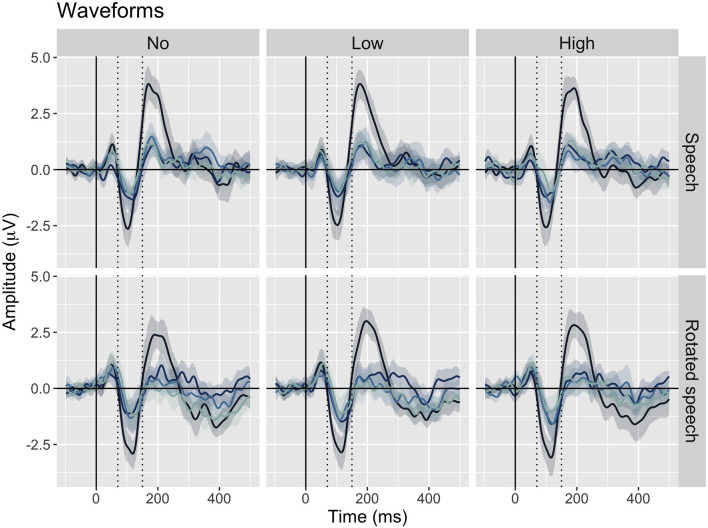
Grand average waveforms at Cz for each position in stimulus trains (position 1–4, from dark to light blue), sorted by speech type condition (rows) and variability condition (columns). Negativity is plotted down. A prominent N1 is apparent between 70 and 150 ms (dashed lines) in all conditions.

**Figure 2:**
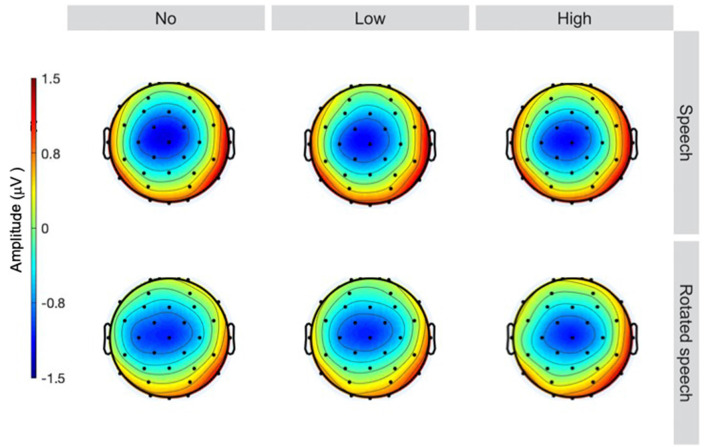
Topographies at 90 ms, sorted by speech type condition (rows) and variability condition (columns). A centrally distributed N1 is apparent in all conditions (head seen from above, nose up).

The averaged ERPs show prominent N1s at expected latencies (Figure 1) and scalp locations (Figure 2) in all speech type and variability conditions (vowel conditions pooled for illustration purposes).

**Figure 3:**
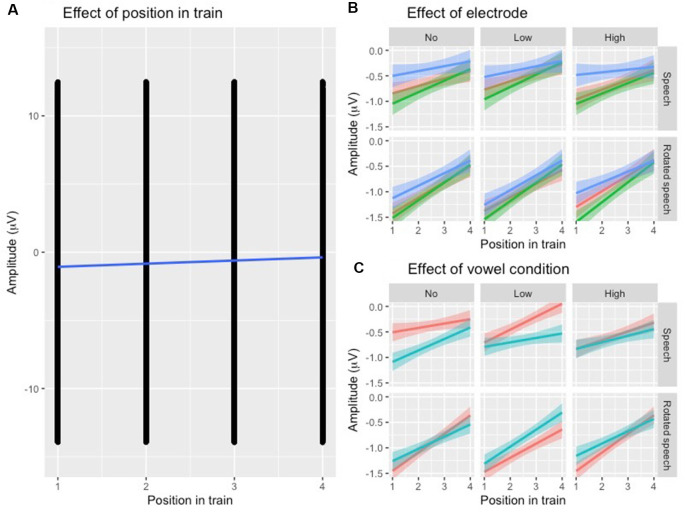
**(A)** Scatterplot of all data points with the position in the stimulus train on the x-axis and N1 amplitude (μV) at the y-axis. The blue regression line shows that repetition-attenuation of the N1 amplitude occurs. **(B)** Illustration of N1 repetition-attenuation as a function of electrode (red = C3, green = Cz, blue = C4), across speech and variability conditions. **(C)** Illustration of N1 repetition-attenuation as a function of vowel condition (red = /e/, blue = /i/), across speech and variability conditions.

To establish that N1 repetition-attenuation occurred, the relationship between N1 amplitude and position in the stimulus trains was investigated using linear mixed-effects regressions. The predicted variable was N1 AMPLITUDE, fixed effects were POSITION, SPEECH TYPE CONDITION, VARIABILITY CONDITION, VOWEL CONDITION and ELECTRODE, and random effects were intercepts for SUBJECT and TRAIN, as well as by-subject and by-train random slopes for the effect of POSITION. The model revealed that the amplitude became more positive with an average of 0.24 μV (95% CI 0.14 μV | 0.34 μV) for each stimulus presentation within a train (Table 2, Figure 3A), that is, it was established that N1 repetition-attenuation was present. Next, an inspection of the pattern of attenuation by speech and variability conditions reveals that both the impact of electrode (Figure 3B) and the impact of vowerl condition (Figure 3C) appear quite disparate for different experimental conditions (SPEECH TYPE CONDITION, VARIABILITY CONDITION). Based on this, interactions between those conditions and the experimental conditions are considered in the main analysis.

**Table 2:** Summary of the fixed effects of the preliminary analysis.

Fixed effects	Est.	SE	*t*
Intercept (position 1, speech, LoVar, /e/, C3)	−1.17	0.22	**−5.30**
Position	0.24	0.05	**4.74**
Speech type condition (rotated speech)	−0.34	0.03	**−11.0**
Variability condition (HiVar)	−0.01	0.04	−0.19
Variability condition (NoVar)	−0.01	0.04	−0.33
Vowel condition (/i/)	−0.06	0.03	−1.82
Electrode (Cz)	−0.07	0.04	**−1.96**
Electrode (C4)	0.20	0.04	**5.24**

*According to the t-as-z approach to estimate statistical significance (threshold ±1.96; Luke, 2017), the effects of position, speech type condition, and electrode can be considered significant (bolded)*.

**Figure 4:**
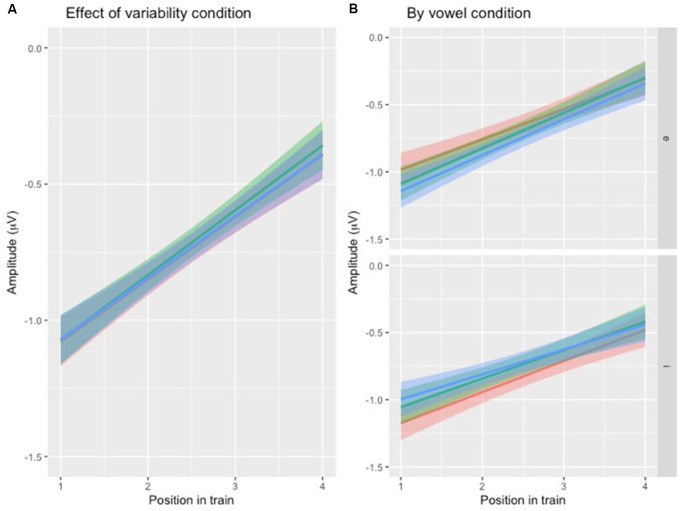
**(A)** Illustration of N1 repetition-attenuation as a function of variability condition (red = no, green = low, blue = high). Note that all three variability conditions are included in the plot but that lines overlap, potentially making them hard to distinguish. **(B)** Illustration of N1 repetition-attenuation as a function of variability condition (red = no, green = low, blue = high) by vowel condition.

To test the first hypothesis (Table 1, hypothesis 1), a new model was created, with the same factors as the one above, except that the interaction between position and VARIABILITY CONDITION was included (Table 3, Figure 4A). There was no significant effect of interaction between POSITION and VARIABILITY CONDITION, indicating that the pattern of N1 repetition-attenuation was similar across all three variability conditions when looking at the two speech type conditions combined.

**Table 3:** Summary of the fixed effects of the variability analysis.

Fixed effects	Est.	SE	*t*
Intercept (position 1, speech, LoVar, /e/, C3)	−1.17	0.23	**−5.20**
Position	0.24	0.05	**4.47**
Speech type condition (rotated speech)	−0.34	0.03	**−11.0**
Variability condition (HiVar)	−0.01	0.09	0.08
Variability condition (NoVar)	0.01	0.09	0.11
Vowel condition (/i/)	−0.06	0.03	−1.82
Electrode (Cz)	−0.07	0.04	**−1.96**
Electrode (C4)	0.20	0.04	**5.24**
Position: variability condition (HiVar)	<0.01	0.03	<0.01
Position: variability condition (NoVar)	0.01	0.03	−0.26

*The same main effects as in the preliminary analysis are considered significant (bolded). The interaction between position and variability condition was not significant*.

Following up on the visual impression of an effect of electrode (Figure 3B), a likelihood ratio test was performed to compare the model above (Table 3) with one in which ELECTRODE was added to the interaction under investigation. This revealed that the three-way interaction between position, VARIABILITY CONDITION and ELECTRODE was not significant ($\chi_{\left(10\right)}^{2}$ = 17.99, *p* = 0.055). The same procedure was used to follow up on the visual impression of the vowel condition (Figure 3C). This likelihood ratio test revealed that the three-way interaction between POSITION, VARIABILITY CONDITION and VOWEL CONDITION was significant ($\chi_{\left(5\right)}^{2}$ = 12.17, *p* = 0.033, Figure 4B).

**Figure 5:**
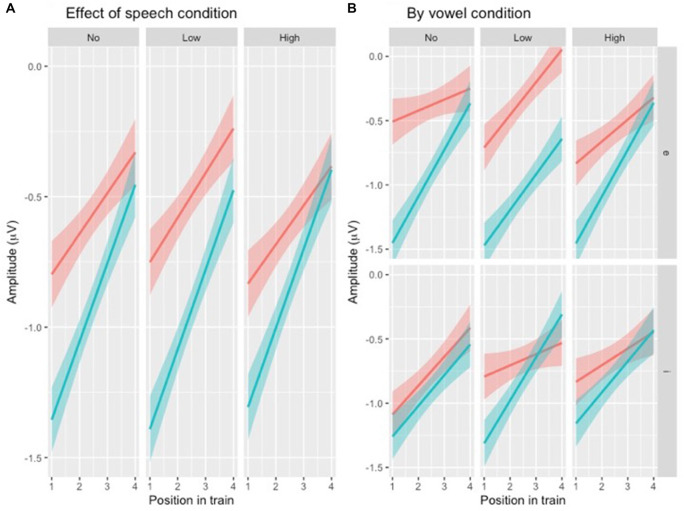
**(A)** Illustration of N1 repetition-attenuation as a function of speech type condition (red = speech, blue = rotated speech) across variability conditions. **(B)** […] (red = speech, blue = rotated speech) by variability and vowel conditions.

To test the hypotheses regarding speech type condition (Table 1, hypotheses 2a-c), the dataset was divided by variability condition and three separate models were created. The factors were identical to the one above except that VARIABILITY CONDITION was not included and the interaction between POSITION and SPEECH TYPE CONDITION was included (Table 4) instead of the one between POSITION and VARIABILITY CONDITION. A similar pattern was found in all three subsets (Figure 5A), with the main effect of SPEECH TYPE CONDITION and its interaction with POSITION being significant in all three variability conditions.

**Table 4:** Summary of the fixed effects of the speech type condition analysis.

	Est.	SE	*t*
A. Fixed effects: NoVar
Intercept (position 1, speech, /e/, C3)	−0.94	0.23	**−4.07**
Position	0.15	0.06	**2.35**
Speech type condition (rotated speech)	−0.70	0.13	**−5.27**
Vowel condition (/i/)	−0.18	0.06	**−3.15**
Electrode (Cz)	−0.05	0.06	−0.82
Electrode (C4)	0.24	0.06	**3.71**
Position: speech type condition (rotated speech)	0.16	0.05	**3.27**
B. Fixed effects: LoVar			
Intercept (position 1, speech, /e/, C3)	−0.98	0.24	**−4.08**
Position	0.18	0.06	**2.98**
Speech type condition (rotated speech)	−0.78	0.13	**−5.85**
Vowel condition (/i/)	−0.04	0.06	−0.75
Electrode (Cz)	−0.05	0.06	−0.77
Electrode (C4)	0.16	0.06	**2.47**
Position: speech type condition (rotated speech)	0.14	0.05	**2.82**
C. Fixed effects: HiVar			
Intercept (position 1, speech, /e/, C3)	−1.08	0.26	**−4.12**
Position	0.16	0.07	**2.32**
Speech type condition (rotated speech)	−0.58	0.13	**−4.30**
Vowel condition (/i/)	0.04	0.06	0.80
Electrode (Cz)	−0.12	0.06	−1.88
Electrode (C4)	0.20	0.06	**3.04**
Position: speech type condition (rotated speech)	0.14	0.05	**2.94**

*A: NoVar dataset. The same main effects as in previous analyses are significant (bolded), but also so are both the main effect of vowel condition and the interaction between position and speech type condition. B: LoVar dataset. The same main effects as in previous analyses are significant (bolded), as is the interaction between position and speech type condition. C: HiVar dataset. The same main effects as in previous analyses are significant (bolded), but also so is the interaction between position and speech type condition*.

For each subset, the above model (Table 4) was compared to one in which VOWEL CONDITION was added to the interaction. For NoVar and LoVar, the addition proved significant (NoVar: $\chi_{\left(3\right)}^{2}$ = 20.09, *p* < 0.001; LoVar: $\chi_{\left(3\right)}^{2}$ = 33.88, *p* < 0.001), but for HiVar it was not significant ($\chi_{\left(3\right)}^{2}$ = 7.676, *p* = 0.053), see Figure 5B.

To summarize, the repetition-attenuation of the N1 amplitude did occur but was not impacted by the variability condition. It was however impacted by speech type condition, with more attenuation found for rotated speech than for speech in all variability conditions. For a summary of the findings as compared to our hypotheses, see Table 5. Last but not least, we found an unexpected effect of vowel condition interacting with both variability condition and speech type conditions to impact the pattern of attenuation.

**Table 5:** Summary of our predictions and the results.

#	Prediction	Results	Data
1	NoVar > LoVar > HiVar	NoVar = LoVar = HiVar	Speech and rotated speech pooled
2a	Speech > Rotated speech	Rotated speech > Speech	NoVar
2b	Speech > Rotated speech	Rotated speech > Speech	LoVar
2c	Speech = Rotated speech (no N1 suppression)	Rotated speech > Speech	HiVar

*The degree of attenuation (steepness of regression line across position in the train) is ranked*.

## Discussion

Findings demonstrate N1 repetition-attenuation both for the exact repetition of stimuli and in the face of acoustic and perceptual category variability. The overall degree of attenuation did not differ as a function of variability. This is contrary to our first prediction, and as such, our findings do not support the refractoriness hypothesis of N1 repetition-attenuation (Budd et al., 1998; Rosburg et al., 2010). Nor are they in line with the predictive coding hypothesis of N1 repetition-attenuation (Hsu et al., 2016), since no correspondence was found between predictability of the stimuli (a measure of predictability in NoVar, no predictability in LoVar and HiVar) and degree of attenuation (no difference between variability conditions).

The findings are however in line with previous findings suggesting that the repetition-attenuation of the N1 subcomponent under investigation here (N1b) is general in nature and not closely tied to the spectrotemporal characteristics of the stimuli. Leung et al. (2013) found no difference in N1 repetition-attenuation between trials comprised of the same or different types of non-speech sounds. Similarly, the findings of the present study suggest that N1 repetition-attenuation does not reflect the degree of acoustic or perceptual overlap between stimuli. Of interest for future studies would be to consider other N1 subcomponents, such as the temporal N1a and N1c (e.g., Woods, 1995; Zhang et al., 2011). Since different subcomponents reflect different processes (Näätänen and Picton, 1987) and to some extent appear differently sensitive to repetition-attenuation (Zhang et al., 2011), they may be impacted to varying degrees by acoustic and perceptual category variability and overlap.

Regarding the relative attenuation for speech and rotated speech, the findings were not in line with our predictions. We predicted that speech would show more attenuation in NoVar and LoVar since the total of repetition can be considered greater in speech (acoustic plus category repetition) than in rotated speech (acoustic repetition only). The findings reveal instead more attenuation for rotated speech than for speech in both the NoVar and the LoVar condition. In the HiVar condition, we predicted that little or no N1 attenuation would be demonstrated in either speech type condition. Contrary to this, substantial N1 attenuation was demonstrated both in speech and in rotated speech, and contrary to our prediction more attenuation was demonstrated for rotated speech than for speech.

Our findings are in line with those of Teismann et al. (2004), who found less attenuation for speech than for non-speech, albeit only in the left hemisphere. Contrary to Teismann’s findings, however, no difference between hemispheres was apparent for the two speech type conditions in the present study. This can potentially be explained by the fact that electroencephalography, used in the present study, does not have the same spatial resolution as magnetoencephalography, used in Teismann’s study (2004). Another possible explanation is that stimuli in the speech type conditions were of comparable acoustic complexity in the present study, whereas the non-speech was considerably less complex in the previous study (Teismann et al., 2004).

Woods and Elmasian (1986) instead found more attenuation for speech than for non-speech. Their non-speech stimuli were less complex than their speech stimuli, but importantly their speech stimuli consisted both of vowels and of CVC syllables that were also lexical items, a fact that is likely to have impacted the results. Yue et al. (2017) compared N1 repetition-attenuation to single-syllable words and phonologically matched nonsense words, demonstrating that the lexical status of the stimuli impacts N1 attenuation. However, their findings are in the opposite direction of those of Woods and Elmasian. While Woods and Elmasian found more attenuation for their speech stimuli (including lexical items), Yue et al. (2017) instead reported less repetition-attenuation in response to their lexical items. It is of course possible that if separated, the two speech conditions of Woods and Elmasian would show the same pattern, but that particular comparison is not reported (Woods and Elmasian, 1986). It is also important to keep in mind that the measure in Woods and Elmasian (1986) was not attenuation of N1 only, but rather an attenuation of the N1-P2 complex, which makes direct comparisons with this and other previous studies on N1 repetition-attenuation in response to speech and non-speech stimuli somewhat problematic.

To summarize, the findings of the present study show that the degree of acoustic and perceptual overlap does not impact the degree of N1 attenuation. While we do find that speech sounds are less attenuated than non-speech sounds, it is not clear whether or not this is related specifically to the categorized perception of speech or some other speech processing characteristic.

An unexpected finding was that the specific vowel condition significantly impacted the pattern of attenuation for both experimental conditions (Figures 3C, 4B, 5B). Possible explanations for this include the relative prototypicality of the different vowel exemplars along the /i/-/e/ continuum, and the relative propensity of the rotated vowels to in some cases be perceived as vowels rather than non-speech sounds—in particular considering the repetitive context in which the sounds were presented. Differences in exemplar prototypicality for the two vowel conditions could potentially lead to differences in the speech condition across all three variability conditions. However, assuming that the finding of diminished attenuation for speech relative to non-speech has to do with perceptual category processing, different degrees of attenuations are to be expected as a result of differences in prototypicality rather than differences in absolute amplitudes. The difference in absolute amplitudes is rather expected due to the vowels’ acoustic differences, especially as it is not present in the conditions where variability was present (LoVar and HiVar), but not in the condition with repetition of identical stimuli (NoVar). Due to the basic acoustic complexity and structure being maintained in the rotation procedure, rotated vowels can be similar enough to real vowels for them to be perceived as such. The vowels in the present study were of course selected so as not to greatly resemble any existing Swedish vowels, but considering the long duration of the experiment in combination with the repetitive presentation of short vowel-like sounds, possibly the rotated vowels at times were perceived as odd exemplars of vowels (just like isolated real vowels presented repeatedly in a long experiment can start to be perceived as mechanical noise or buzzing). Assuming that this type of auditory “drift” occurred to some extent for both vowels and rotated vowels, a difference in exactly how close to real vowels the rotated vowels are acoustical could explain differences in the pattern of attenuation. For example, if the rotated /i/ is more susceptible to being perceived as a vowel rather than a non-speech sound than the rotated /e/ is, and/or the /i/ is more susceptible than the /e/ to being perceived as noise, this would explain the differences in attenuation in the NoVar condition, where the attenuation-patterns for /i/ and rotated /i/ are more similar than those of /e/ and rotated /e/. This potential concern can in future studies be alleviated by having participants perform perceptual identification and/or discrimination of the stimuli to account for individual speech sound category boundaries. The present study would of course benefit from replication, preferably with additional vowels to further explore this particular issue.

In conclusion, the present study demonstrates N1 repetition-attenuation to speech and non-speech stimuli in the face of acoustic and perceptual category variability, but no relationship between the degree of variability and degree of attenuation is evidenced. Speech resulted in less attenuation than acoustically comparable non-speech, in line with findings from previous studies with comparable methods and stimuli (Teismann et al., 2004).

## Data Availability Statement

Additional information and tabular data generated for this study are available at the Open Science Framework (https://osf.io/fgwe6/).

## Ethics Statement

The studies involving human participants were reviewed and approved by the National Swedish Ethics Board (2019-00685). The participants provided their written informed consent to take part in this study.

## Author Contributions

All authors contributed to the article and approved the submitted version. EM: study conceptualization, stimuli, and experiment creation. EM, ICS, LG, and PK: study design, data collection, drafting the manuscript, and critical revisions to the manuscript. EM and PK: data preprocessing.

## Conflict of Interest

The authors declare that the research was conducted in the absence of any commercial or financial relationships that could be construed as a potential conflict of interest.

## Footnotes

^1^It is well-established that multiple processes contribute to the N1 wave (e.g., Näätänen and Picton, 1987). The present study focuses on the N1b subcomponent, which is most prominent at central electrode sites and peaks at around 100 ms (e.g., Woods, 1995; Zhang et al., 2011). Henceforth “N1” refers to the N1b, unless otherwise specified.

^2^Additional information and data is made available on the OSF site https://osf.io/fgwe6/
